# The hemoglobin Gly16β1Asp polymorphism in turbot (*Scophthalmus maximus*) is differentially distributed across European populations

**DOI:** 10.1007/s10695-020-00872-y

**Published:** 2020-10-04

**Authors:** Øivind Andersen, Juan Andrés Rubiolo, Maria Cristina De Rosa, Paulino Martinez

**Affiliations:** 1 Nofima, PO Box 5010, N-1430 Ås, Norway; 2 Department of Animal and Aquacultural Sciences (IHA), Faculty of Life Sciences (BIOVIT), Norwegian University of Life Sciences (NMBU), PO Box 5003, 1433 Ås, Norway; 3 Department of Zoology, Genetics and Physical Anthropology, University of Santiago de Compostela, Lugo, Spain; 4 Institute of Chemical Sciences and Technologies “Giulio Natta” (SCITEC) – CNR c/o Catholic University of Rome, 00168 Rome, Italy

**Keywords:** Turbot, Hemoglobin, Polymorphism, Body growth, Genetic variation, Adaptation

## Abstract

Turbot is an important flatfish widely distributed along the European coasts, whose fishery is centered in the North Sea. The commercial value of the species has boosted a successful aquaculture sector in Europe and China. Body growth is the main target of turbot breeding programs and is also a key trait related to local adaptation to temperature and salinity. Differences in growth rate and optimal growth temperature in turbot have been shown to be associated with a hemoglobin polymorphism reported more than 50 years ago. Here, we identified a Gly16Asp amino acid substitution in the β1 globin subunit by searching for genetic variation in the five functional globin genes within the whole annotated turbot genome. We predicted increased stability of the turbot hemoglobin by the replacement of the conserved Gly with the negative charged Asp residue that is consistent with the higher rate of αβ dimer assembly in the human J-Baltimore Gly16β->Asp mutant than in normal HbA. The turbot Hbβ1-Gly16 variant dominated in the northern populations examined, particularly in the Baltic Sea, while the Asp allele showed elevated frequencies in southern populations and was the prevalent variant in the Adriatic Sea. Body weight did not associate with the Hbβ1 genotypes at farming conditions (i.e., high oxygen levels, feeding ad libitum) after analyzing 90 fish with high growth dispersal from nine turbot families. Nevertheless, all data at hand suggest that the turbot hemoglobin polymorphism has an adaptive significance in the variable wild conditions regarding temperature and oxygen availability.

**Electronic supplementary material:**

The online version of this article (10.1007/s10695-020-00872-y) contains supplementary material, which is available to authorized users.

## Introduction

Flatfish represent a highly specialized teleost group adapted to demersal life, and the rather inactive lifestyle and low metabolism allow flatfish in general to cope with fluctuating temperatures and oxygen levels in shallow water habitats. However, interspecific differences have been found in the tolerance to high temperatures and hypoxic conditions that seem to be associated with differences in gill ventilation, oxygen uptake, and functional properties of hemoglobins (Weber and de Wilde 1975, 1976; Steffensen et al. 1982; van den Thillart et al. 1994; Taylor et al. 2007). Moreover, larval dispersal and connectivity among populations were recently shown to vary among six flatfish species in the Northeast Atlantic Ocean (Barbut et al. 2019), and local adaptation and genetic divergence between populations have consistently been reported in several species, including common sole (*Solea solea*) (Diopere et al. 2018), European flounder (*Platichthys flesus*) (Hemmer-Hansen et al. 2007; Pédron et al. 2017), and turbot (*Scopthalmus maximus*) (Vandamme et al. 2014; Vilas et al. 2015; do Prado et al. 2018a).

The turbot (*Scophthalmus maximus*) is widely distributed along the coast of the Northeast Atlantic Ocean from North Africa and the Mediterranean Sea in the south to the Icelandic Sea and the Western Norwegian Sea in the north. This flatfish species also occurs in the Baltic Sea and in the Black Sea, two inner low-salinity seas, where parallel evolution related to salinity has been recently suggested (do Prado et al. 2018a). Although the Black Sea turbot was initially claimed to be a separate species (*S. maeoticus*) based on morphological features, population genetic data support its status as a subspecies (*S. maximus maeoticus*) (Bouza et al. 1994; do Prado et al. 2018a). Turbot is a rather stationary species, although adults can move kilometers offshore to deeper waters likely related to spawning (Bergstad and Folkvord 1997; Bouza et al. 1994). Due to its high commercial and nutritional value, turbot is an important cultured species in several European countries and has been introduced into the Southeast Pacific Ocean (Chile) and China for farming. Evaluation of genetic variation and selection for increased growth rates, disease resistance, and temperature tolerance are the main targets of turbot breeding programs (Martínez et al. 2016; Wang et al. 2019). Optimal temperature for growth has been reported to range from 13 to 23 °C depending on size (Burel et al. 1996; Imsland et al. 1996, 2000a; Árnason et al. 2009). Interestingly, juvenile turbot from a Norwegian population were found to have higher optimal temperature for growth and feed conversion efficiency than fish from Scottish and French populations in a common garden experiment (Imsland et al. 2000b). Differences in growth performance were suggested to be at least partly due to adaptation to different temperatures regimes at high and low latitudes affecting metabolic rates and oxygen availability that in turn could involve the polymorphic turbot hemoglobin (Imsland et al. 1997; Imsland et al. 2000a; Samuelsen et al. 1999).

Turbot hemoglobin polymorphism was originally described by Manwell and Baker (1967, 1970) by electrophoretic separation of two homozygous phenotypes possessing a fast (β^F^) or slow (β^S^) migrating β subunit and a heterozygous type displaying both variants. Samuelsen et al. (1999) studied the oxygen-binding properties of the three genotypes designated Hb-I(1/1), Hb-I(2/2), and Hb-I(1/2) of which the latter exhibited the highest oxygen-binding affinity followed by Hb-I(1/2) and Hb-I(1/1) within a temperature range of 10–19 °C. Hb-I(2/2) fish were suggested to have an adaptive advantage over the other genotypes under certain environmental conditions based on the higher specific growth rates and higher optimal temperature for growth shown in controlled experiments (Samuelsen et al. 1999; Imsland et al. 2000a). An initial approach to the distribution of the Hb-I alleles in the Northern Atlantic populations showed that Hb-I(1) dominated in this region, but its frequency was significantly higher in the Baltic Sea, Kattegat, and Southwest Norway than in Iceland and West Norway (Imsland et al. 2003).

The availability of thousands of single nucleotide polymorphisms (SNP) covering the whole length of the consistently annotated turbot genome (Figueras et al. 2016; Maroso et al. 2018) facilitates a deeper characterization of synonymous and non-synonymous variation in candidate genes to examine their involvement in local adaptation. Furthermore, the detailed population genomics picture reported for the species across its full distribution range (do Prado et al. 2018a), offers the opportunity for an evaluation of these polymorphisms across the European coast to test hypothesis relating them to environmental variables. This information might be useful for fisheries management and for turbot breeding programs depending on the location of farms across Europe. The objective of this study was to identify and characterize the genetic variation in the turbot globin genes underlying the previously reported hemoglobin polymorphism of the species. Starting from this information, we examined the distribution of the identified Hbβ1 allelic variants across European populations and checked their association with body growth in fast and slow growing families of farmed turbot to ascertain their putative association with adaptation in wild and domestic conditions.

## Results and discussion

### Genomic organization of turbot globin genes

**Fig. 1:**
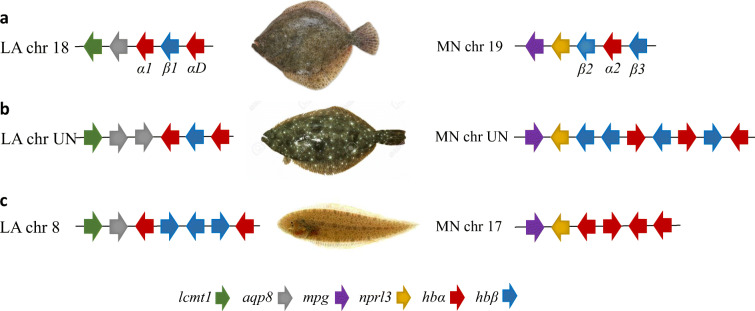
Genomic organization of the LA and MN globin gene clusters in **a** turbot, **b** Japanese flounder, and **c** half-smooth tongue sole. Arrows show the transcriptional direction of the genes. The turbot α and β genes are referred to as indicated. Chr, chromosome; UN, unknown

The turbot genome has been shown to harbor three α globin and three β globin genes, which are organized in two clusters located on chromosomes (chr) 18 and 19 (Fig. 1) (Maroso et al. 2018). The globin clusters are positioned adjacent to the conserved *lcmt1-aqp8* (LA) and *mcp-nprl1* (MN) genes, respectively, in the three available flatfish genomes of turbot, Japanese flounder (*Paralichthys olivaceus*), and half-smooth tongue sole (*Cynoglossus semilaevis*). Both the so-called LA and MN globin clusters contain α and β globin genes in teleosts (Opazo et al. 2013), but exceptions are found, such as the loss of β genes in the tongue sole MN cluster (Chen et al. 2014). The turbot *hbαD*-like gene (AWP17399.1) codes for a predicted protein of only 112 amino acids and is probably a pseudogene, since vertebrate α globins normally consist of about 143 residues.

### Two polymorphic turbot Hbβ globins

**Fig. 2:**
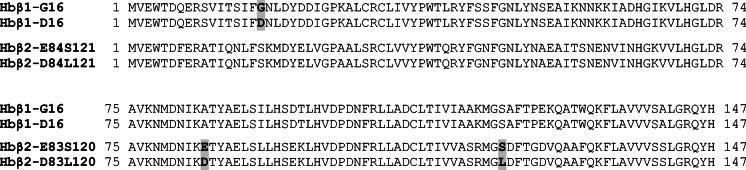
Sequence alignments of the polymorphic Hbβ1 and Hbβ2 globins in turbot. Amino acid substitutions are highlighted and numbered without counting Met at position 1

We searched for genetic variants in the five functional turbot globin genes by re-sequencing 10 individuals originating from 10 unrelated families of a Spanish turbot farm. Two synonymous SNPs were found in the *hbα2* gene (AWP19130.1) on chr 19, while the adjacent *hbβ2* gene (AWP19132.1) was shown to possess two non-synonymous SNPs resulting in the Glu-Asp and Ser-Leu changes at positions 83 and 120, respectively, of the predicted protein. Furthermore, a non-synonymous SNP in the *hbβ1* gene (AWP17400.1) on chr 18 caused a Gly-Asp change at position 16 (Fig. 2). Whereas identical isoelectric point (pI) of 5.67 was calculated for the Hbβ2 variants, the Hbβ1-Gly16 and Hbβ1-Asp16 variants have pI of 6.95 and 6.50, respectively, in compliance with the cathodic Hb-I(1/1) and anodic Hb-I(2/2) types previously identified by isoelectric focusing (Manwell and Baker 1967; Imsland et al. 1997).

**Fig. 3:**
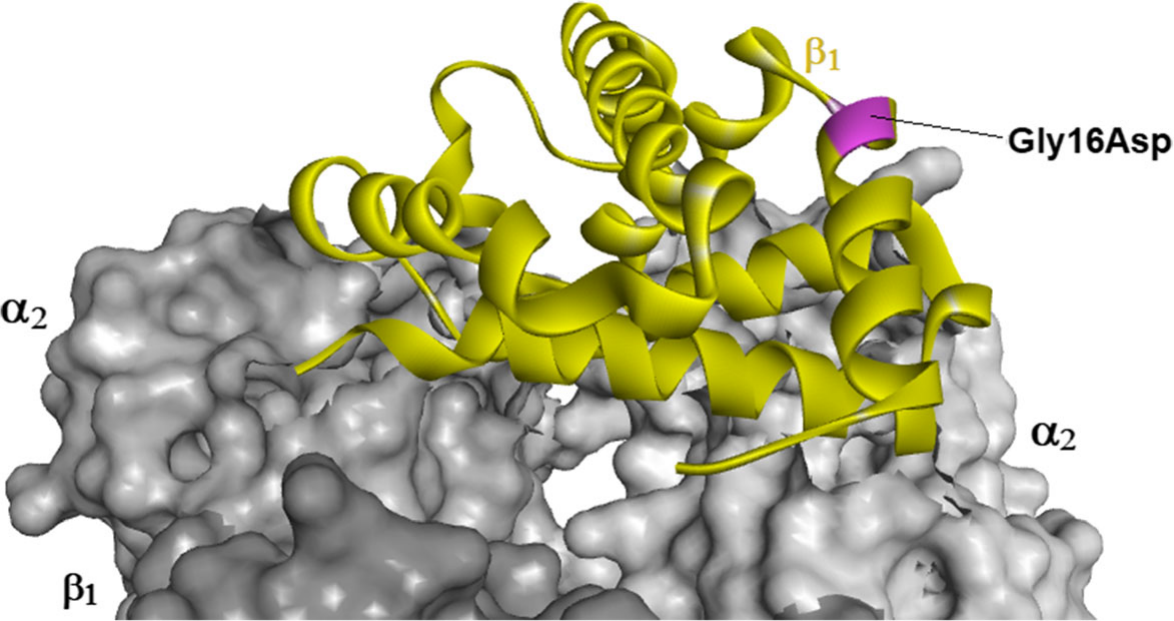
Structural model of the polymorphic turbot Hbβ1 subunit within the hemoglobin tetramer. The Gly16Asp substitution in the A-helix is indicated

The 3D modeling of the Gly16Asp substitution in the Hbβ1 globin revealed no structural changes (Fig. 3). However, the β subunits of the tetrameric hemoglobin are probably stabilized by the Asp replacement, which introduces a carboxylated group facing water at the surface of the protein. Accordingly, a stabilization effect of the replacement was suggested by the mutation energy change of − 1.9 kcal/mol calculated for the Gly16->Asp change in turbot Hbβ1. Notably, a − 1.3 kcal/mol energy stabilization value was found for the human J Baltimore Gly16β->Asp mutant. This Hb variant showed a higher rate of αβ dimer assembly than normal HbA, indicating that the formation of dimers is facilitated by the electrostatic attraction between the charged α and β subunits (Bunn and McDonald 1983; Mrabet et al. 1986). Subunit competition may explain why erythrocytes in human Hb J Baltimore (Gly16β->Asp) and N Baltimore (Gly16β->Glu) mutants often contain more of the mutated variant than that of HbA (Mrabet et al. 1986). Competition between subunit variants may also underlie the reported hybrid of the turbot hemoglobin tetramer in heterozygous fish consisting of both β^F^ and β^S^ subunits (Manwell and Baker 1967; Imsland et al. 1997). It should be noted that the neutral Gly or polar Ser is highly conserved at position 16 in teleost β globins, while the Asp residue was only identified in the air breathing climbing perch (*Anabas testudineus*, XP_026229193) and blunt snouted clingfish (*Gouania willdenowi*, XP_028310257) and in the hypoxia tolerant catfish species *Pangasianodon hypophthalmus* (XP_026785154), *Ictalurus punctatus* (XP_017309923), and *I. furcatus* (AD028060). Contrasting with the higher pH sensitivity (Bohr effect) of the cathodic turbot Hb-I(1/1) allelic variant than Hb-I(2/2) (Samuelsen et al. 1999), cathodic teleost globins in general show low pH sensitivity of importance for securing oxygen transport when blood pH drops during hypoxic conditions (Weber and Jensen 1988; Mendez-Sanchez and Burggren 2017).

### Distribution of the Hbβ1 allelic variants in turbot populations

Starting from the in silico information outlined above, we designed appropriate sets of primers using the turbot genome as reference to validate the five globin SNPs detected in a sample of 90 wild specimens using the Sequenom genotyping methodology (Table S1). Then, genotypes for the different Hb variants were obtained in wild populations (Table S2) and domestic families (Table S3) to check for their consistency and putative association with environment variables and local adaptation.

**Fig. 4:**
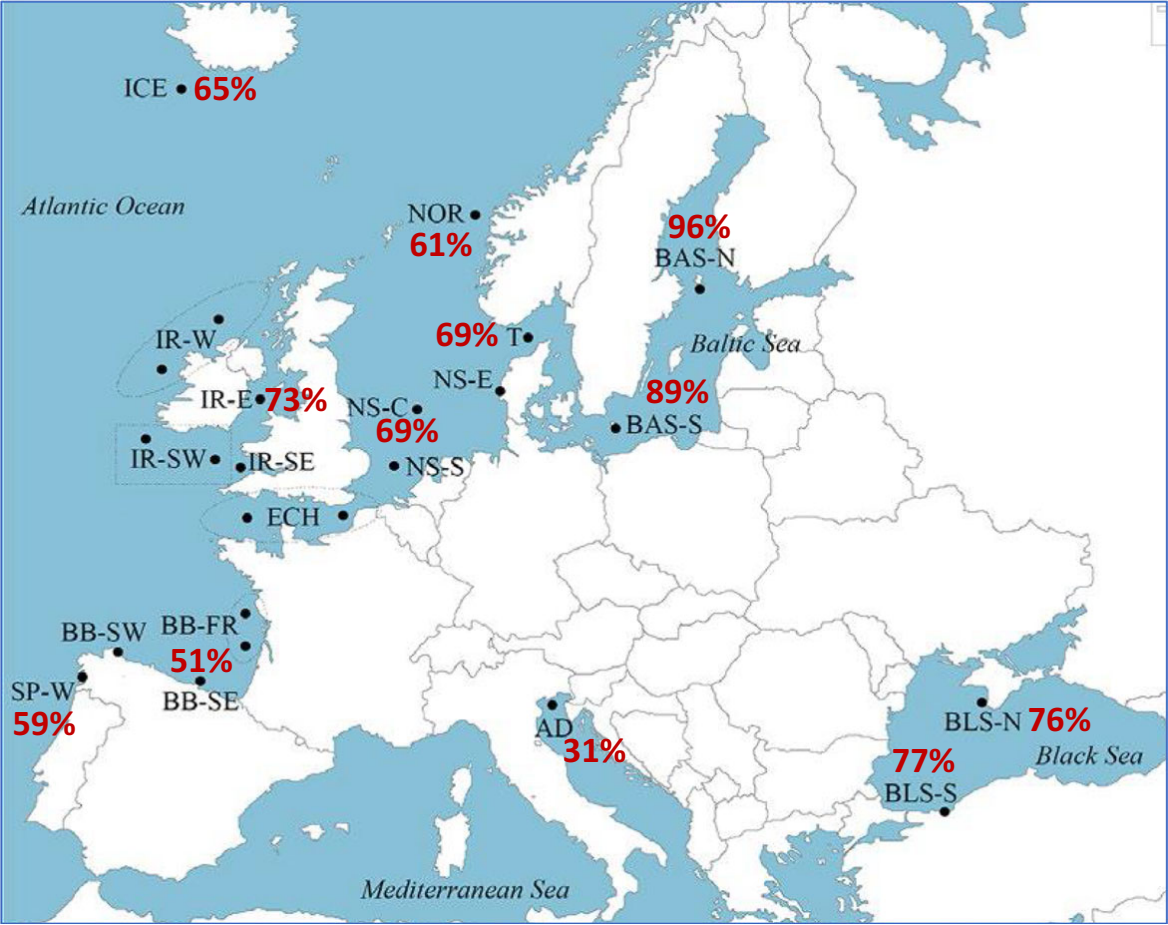
Frequencies of the turbot Hbβ1-Gly16 allele in wild populations throughout the European coasts from the Baltic Sea up to the Black Sea. Details about sample location and sample date are found in do Prado et al. (2018a)

A total of 456 fish sampled from 12 European populations covering the whole turbot distribution were analyzed (Table S2; Fig. 4). While very low polymorphism was detected for the *hbα1* SNPs, medium or high polymorphism was revealed for the three *hbβ* type SNPs. However, the pattern of genotypic variation at the two *hbβ2* SNPs was not consistent either with population or family segregation data, suggesting simultaneous PCR amplification and genotyping of two paralogous genes. Indeed, most populations showed strong deviation from Hardy-Weinberg Equilibrium (HWE) (Fisher’s exact test for all populations *P*_SNP39573_ = 0; *P*_SNP39789_ = 0.009), and no homozygotes for the frequent A and C alleles were detected at the SNP 39573 and SNP 39789, respectively, in any of the populations or families analyzed (Table S2). Furthermore, family segregation was also inconsistent with a single locus, since only heterozygous individuals were detected in the nine families analyzed for the SNP 39573, and again the C homozygotes were not detected in any of the families for the SNP 39789 (Table S3). Conversely, the *hbβ1* polymorphism was fully reliable from population and family data consistent with HWE in all populations (Fisher’s exact test for all populations = 0.963) and Mendelian segregation with family data (Table S4).

We focused on the most interesting and consistent polymorphism at the *hbβ1* gene and estimated the global relative coefficient of differentiation for the 12 populations analyzed (*F*_ST_ = 0.129; *P* = 0) and between all population pairs (*F*_ST_ range: 0.610 between Adriatic Sea and Baltic Sea North—0.000 for many population pairs, particularly involving Iceland and Ireland; Table S5). The global *F*_ST_ (0.129) exceeded the previously reported values with isozymes (0.070; Blanquer et al. 1992) and SNPs (0.090; do Prado et al. 2018a). This unequal distribution of the *hbβ1* variants throughout the European coast suggests a relationship with environmental variation (Fig. 4). The Hbβ1-Gly16 allele was the most common variant in the North Atlantic populations with allele frequencies ranging from 61 to 73%, in agreement with the prevalence of the Hb-I(1) allele in Iceland and west coast of Norway (Imsland et al. 2003). This cathodic variant was almost fixed in the northern population of the Baltic Sea (94%), while the frequency in the southern Baltic population was 89%, very close to the Hb-I(1) frequency of 84% reported by Imsland et al. (2003). Previous microsatellite and SNP data revealed a rapid transition in the genetic composition for specific genomic regions putatively involved in adaptation to salinity between turbot populations from the brackish Baltic Sea and the North Sea (Nielsen et al. 2004; Vilas et al. 2010, 2015; Vandamme et al. 2014; do Prado et al. 2018a). Influence of salinity on the metabolism was shown in turbot acclimated to salinities ranging from 8 to 35‰, and the lowest routine oxygen consumption rates were measured at 8‰ salinity with no significant differences in higher acclimation salinities (Waller 1992). Further, reproductive success and growth differences in turbot between the Atlantic Ocean and the Baltic Sea have been associated with salinity (Nissling et al. 2006). Noteworthy, the polymorphic *hbβ1* gene of Atlantic cod showed strong allelic differences between the Baltic Sea and Kattegat that was suggested to be related to differences in water temperature (Andersen et al. 2009).

Whereas the Hbβ1-Gly16 variant dominates in the North Atlantic turbot populations, both alleles were common in the Spanish West and Biscay Bay populations, and further, the Asp16 variant was the most prevalent in the Adriatic Sea (69%) (Fig. 4). Both globin variants were also identified in the Black Sea in agreement with the reported hemoglobin polymorphism identified by isoelectric focusing (Ivanova et al. 2006). The increased frequencies of the Gly variant in the Black Sea could be related to its lower salinity, suggesting a parallelism with the Baltic Sea as previously reported (do Prado et al. 2018a), despite the temperature regimes that are rather different between the two areas.

### Turbot Hbβ1 polymorphism and body growth

The increased stability predicted for the Hbβ1-Asp16 variant and the increased frequencies southwards in the North Atlantic populations examined suggest an association between its functional properties and temperature. Accordingly, the high oxygen-binding affinity of HbI-(2/2) was suggested to be beneficial at high temperatures with lowered oxygen availability and increased metabolism (Imsland et al. 1997; Samuelsen et al. 1999). However, while the Hb-I(2/2) fish grew significantly faster than the other two genotypes at low (10 °C) and optimal (16 °C) temperatures, no differences in growth were found between the three genotypes at 19 °C (Imsland et al. 1997). We examined the putative association between body growth and the Hbβ1 polymorphic variants at farm conditions in 90 juvenile turbot (mean weight = 33.3 g; range: 10.1–65.5 g) representing nine families (10 individuals/family) by including extreme growth rate fish within each family (five high and five low) (Table S3). No differences in body weight were detected between the Hbβ1-Gly16 and Hbβ1-Asp16 genotypes in the whole sample at 148 days post-hatching (dph) using either a Kruskal-Wallis test considering weight as a continuous variable (*H* = 3.339; *P* = 0.188) or with a Mann-Whitney test considering weight as a qualitative variable (high vs low weight; *P* = 0.934), despite the different functional properties of the two variants regarding oxygen affinity (Samuelsen et al. 1999) and predicted stability of the tetramer. However, our results should be considered as preliminary, since we cannot exclude possible tank effects when families grew separately, but in the same conditions, until 100 dph, and that families were distributed in 36 tanks following a random scheme until 148 dph (Anacleto et al. 2019). It is also possible that the optimal environmental conditions for turbot farming, including high oxygen pressure and ad libitum feeding, may not distinguish the different performance of the two allelic variants supported by observations in wild populations. Thus, we cannot either reject the possibility that differences could occur later at market size. Moreover, the water temperature of 17–18 °C could also explain the lack of significant differences for growth in our trial in view of the previous study by Imsland et al. (1997).

## Materials and methods

### Biological material

Ten individuals were re-sequenced taking the most recent turbot genome assembly as reference (ASM318616v1; GenBank accession GCA_003186165.1; Maroso et al. 2018) and were used to identify SNP variants at the globin genes in turbot. Barcoded libraries of 150 bp paired-end reads, constructed for each individual and subsequently sequenced at an estimated coverage of 20× per individual, were used for it. This information was taken from the study by Martínez et al. (2019), where five males and five females were screened to identify SNP polymorphism associated with sex in turbot.

A total of 458 individuals originating from 12 populations across the European coasts were genotyped to analyze the distribution of the identified Hb SNPs from the North Atlantic Ocean and Baltic Sea, through the Spanish coasts up to the Adriatic and Black seas. DNA samples were taken from the EU AQUATRACE project (no. 311920) collection, where populations across the whole European coast were surveyed for analyzing the impact of aquaculture in wild populations. Only individuals with not any trace of farm introgression were selected for this study (do Prado et al. 2018b).

Association between hemoglobin polymorphism and body growth at farm conditions was examined in nine full-sib families founded within the EU FISHBOOST project no. 613611 in a trial devoted to analyze the genetic basis of resistance to the parasite *P*. *dicentrarchi* (Anacleto et al. 2019). We took advantage of this experiment, where growth conditions were very homogeneous and where tank effects on phenotypes were randomized, to evaluate the association of hemoglobin variants with growth. Nine families, among the 18 available in the first trial, were selected considering their balanced sex ratio and high growth dispersion. SNPs detected in the coding regions of the five turbot globin genes were genotyped in 90 individuals (10 individuals per family) taken from the ends of the body weight (BW) distribution within each family, i.e., the five with highest BW and the five with lowest BW. The families were all maintained at the same conditions during the period from October 2 to January 14. Fish were grown on isolated 50-l closed-circuit aerated tanks with constant water temperature of 17–18 °C and 36 ‰ salinity. Families were maintained in individual tanks until tagging with elastomeres at 100 dph and were then mixed after 48 days following a randomize scheme to avoid tank effects (Anacleto et al. 2019). Fish weight was determined at 148 dph prior to the starting of a challenge experiment related to resistance to *Philasterides dicentrarchi*. Both DNA, family, and phenotypic information were taken from the EU FISHBOOST project.

### Identification and genotyping of turbot Hb polymorphisms

The α and β globin genes are located between 9,100,000–9,130,000 bp and 14,170,000–14,220,000 bp of chromosomes 18 and 19, respectively, in the turbot genome (Maroso et al. 2018). Reads of the ten re-sequenced individuals (Illumina 150 bp PE) matching at those positions were reassembled using SamTools v1.8 (Li 2011) and Bowtie2 v2.3.4.3 (Langmead and Salzberg 2012). Variants were called using SamTools, VcfTools 0.1.16-1 (Danecek et al. 2011), VarScan v2.3.8 (Koboldt et al. 2009), and BedTools v2.29.2 (Quinlan and Hall 2010). An in-house script was used to consistently identify the SNPs producing non-synonymous variants in the β globin genes based on the coverage and the frequency of allelic variants within and between individuals. SNPs detected in the coding regions of the α- and β-globin genes by both VcfTools and VarScan were genotyped and validated using a MassARRAY platform (Sequenom, San Diego, CA, USA) following the protocols and recommendations provided by the manufacturer. Briefly, the technique consisted of an initial locus-specific polymerase chain reaction (PCR), followed by a single-base extension using mass-modified dideoxynucleotide terminators of an oligonucleotide primer that anneals immediately upstream of the polymorphic site (SNP) of interest. The distinct mass of the extended primer identifies the SNP allele. Primer sequences, SNP position, and expected variants of the SNPs tested are shown on Table S1. MALDI-TOF mass spectrometry analysis in an Autoflex spectrometer was used for allele scoring.

### Molecular modeling

Homology modeling was performed using the Discovery Studio v.19 (Dassault Systems) software suite. The turbot Hbα2 and Hbβ1 target sequences were subjected to BLAST search (https://blast.ncbi.nlm.nih.gov/Blast.cgi) for template identification. ClustalW v2.1 (https://www.genome.jp/tools-bin/clustalw) was used for sequence alignment with the template structures identified in the α chain of the Antarctic fish (*Trematomus newnesi*) hemoglobin (pdb: 3NFE, 65% sequence identity, Vergara et al. 2010) and in the β chain of the cathodic hemoglobin component from the same species (pdb: 2AA1, 67% sequence identity, Mazzarella et al. 2006). Generation of the quaternary structure was based on the program Modeller (Sali and Blundell 1993) and the Discovery Studio software as reported in detail (Verde et al. 2005). The tetramer structure was further validated using Procheck (Laskowski et al. 1993), which has shown that 94.5% of the residues fall in the core region and 5.5% in the allowed region, and Verify3D (Lüthy et al. 1992) which has revealed that 91.8% of the residues have averaged 3D-1D score over 0.2. The Calculate Mutation Energy protocol in the Discovery Studio was used to evaluate the effect of the Gly16->Asp replacement on the stability of the turbot Hbβ1 and the human Hb J Baltimore tetramer.

### Population genetics of the globin non-synonymous SNP variants

Departure from Hardy-Weinberg Equilibrium (HWE) was tested in each population using GENEPOP v4.0 (Rousset 2008). Pairwise *F*_ST_ and global *F*_ST_ values between samples were estimated with the same program using 10,000 permutations to test for significance.

### Association with growth at farm conditions

Association between genotypes and weight at farm conditions (90 samples from nine families) was performed using the non-parametric ANOVA (Kruskal-Wallis) at population level (pooling all data in a single population) and with a U Mann-Whitney using a qualitative trait within families (high vs low growth) was performed in the SSPS statistical package (https://www.ibm.com/es-es/analytics/spss-statistics-software).

## Electronic supplementary material

ESM 1 (DOCX 15 kb)

ESM 2 (XLSX 27 kb)

ESM 3 (XLSX 15 kb)

ESM 4 (XLSX 14 kb)

ESM 5 (XLSX 23 kb)
