# Supplementary material for: The hemoglobin Gly16β1Asp polymorphism in turbot (Scophthalmus maximus) is differentially distributed across European populations
Source: Fish Physiol Biochem. 2020 Oct 4;46(6):2367–76. doi: 10.1007/s10695-020-00872-y (PMC7584550; doi:10.1007/s10695-020-00872-y)
Supplement: Supplementary file 1 — (DOCX 15 kb) [file 10695_2020_872_MOESM1_ESM.docx]

**Table S1**: Primers and characteristics of the amplicons for Sequenom genotyping.
